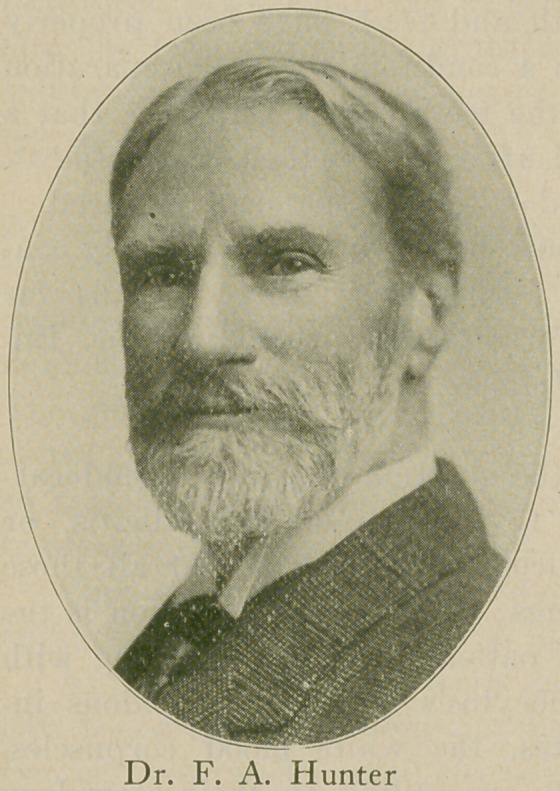# Dr. Frank A. Hunter Honored

**Published:** 1917-04

**Authors:** 


### DR. FRANK A. HUNTER HONORED

On the evening of March 1 Dr. Frank A. Hunter, of Cincinnati, entertained at dinner about thirty of his professional confreres at his delightful home in Mount Auburn. The occasion was the celebration of the fiftieth anniversary of a continuous practice in Cincinnati. Dr. Hunter graduated from the Philadelphia Dental College in 1867, and located immediately in his native city. His father, Dr. Wm. M. Hunter, was a well-known practitioner in Cincinnati for many years. Dr. Frank inherited much of his father's genius and is one of the best all-around dentists Ohio has ever produced. He excells in technical skill, and has always taken great interest in dental education and professional activities. He has a rare personality, which, with his excellent capacity for establishing friendly relations with people, has enabled him to command a practice of unusual character. It was never necessary for him to adopt unusual methods to attract practice. In fact, he has always had a picked practice of the most desirable character. In fact, we know of few men who have honored the profession by so commendable and exemplary professional careers. He has always stood for the highest ideals in his professional and civic life, and is one of the men of real ability and character that is most appreciated where he is best known. Because of inbred modesty, and

Dr. F. A. Hunter not for lack of capability, he has never been a professional office holder nor a prolific writer on professional topics. He possesses peculiar gifts which would cause him to adorn any such opportunities; but for some reason best known to himself, perhaps, he has kept aloof from all public affairs which would in any way tend to make himself more conspicuous. For many years he has been an active member and contributor to the Cincinnati Literary Society, which society has honored him several times as its presiding officer. He was a very close friend of Dr. C. M. Wright, and they enjoyed a remarkable intimacy and companionship. By nature they had much in common, both intellectually smart, and appreciative of good wit.

Several members of the profession from other parts of the State, as well as the Cincinnati men, attended the dinner. After-dinner speeches were made by Doctors Henry Barns, of Cleveland; L. E. Custor, of Dayton; C. I. Keely, of Hamilton, and Doctors F. W. Sage, Walter Stewart, W. S. Locke, J. S. Cassidy, C. H. Schott, H. MacMillan, H. T. Smith, A. G. Rose, T. I. Way and others of the city. Dr. J. R. Callahan, on behalf of Dr. Hunter's Cincinnati friends, presented to him a beautiful silver vase, appropriately inscribed, in testimony of the sincere regard and esteem of his professional associates. Dr. Hunter was much pleased and made a characteristic speech which made everyone present feel that this was a rare occasion and its influence will long remain a precious memory and be a continual reminder that few things in life's experiences are comparable to the making of choice friends. We are sure the many friends and acquaintances of Dr. Hunter who could not participate on this delightful occasion will join in wishing him continued prosperity and happiness on the second lap of his century run.

## Figures and Tables

**Figure f1:**